# An integrative framework for the identification of double minute chromosomes using next generation sequencing data

**DOI:** 10.1186/1471-2156-16-S2-S1

**Published:** 2015-04-23

**Authors:** Matthew Hayes, Jing Li

**Affiliations:** 1 Department of Computer Science, Tennessee State University, 3500 John A. Merritt Blvd, 37209 Nashville, Tennessee, USA; 2 Electrical Engineering and Computer Science, Case Western Reserve University, 10900 Euclid Avenue, 44106 Cleveland, Ohio, USA

**Keywords:** amplicon, double minute, next generation sequencing

## Abstract

**Background:**

Double minute chromosomes are circular fragments of DNA whose presence is associated with the onset of certain cancers. Double minutes are lethal, as they are highly amplified and typically contain oncogenes. Locating double minutes can supplement the process of cancer diagnosis, and it can help to identify therapeutic targets. However, there is currently a dearth of computational methods available to identify double minutes. We propose a computational framework for the idenfication of double minute chromosomes using next-generation sequencing data. Our framework integrates predictions from algorithms that detect DNA copy number variants, and it also integrates predictions from algorithms that locate genomic structural variants. This information is used by a graph-based algorithm to predict the presence of double minute chromosomes.

**Results:**

Using a previously published copy number variant algorithm and two structural variation prediction algorithms, we implemented our framework and tested it on a dataset consisting of simulated double minute chromosomes. Our approach uncovered double minutes with high accuracy, demonstrating its plausibility.

**Conclusions:**

Although we only tested the framework with three programs (RDXplorer, BreakDancer, Delly), it can be extended to incorporate results from programs that 1) detect amplified copy number and from programs that 2) detect genomic structural variants like deletions, translocations, inversions, and tandem repeats.

The software that implements the framework can be accessed here: https://github.com/mhayes20/DMFinder

## Introduction

Double minute chromosomes (DM) are circular fragments of extrachromosomal DNA [1]. They have been found in human tumors of the lungs, ovaries, colon, and breast [2]. They have also been detected in tumors of patients afflicted with neuroblastoma [3]. Double minutes tend to be highly amplified, and they usually contain genes that encode proteins which are essential to cancer formation (oncogenes); these genes may also be highly resistant to drug treatment [4]. The combination of oncogenes and amplification suggests that double minutes are highly lethal, and it is thus important to have efficient methods to locate and characterize them. Such methods could, for example, help researchers to develop drugs that target double minutes. Also, if double minutes are detected, it could help researchers assess the effectiveness of *existing *drugs in the treatment of double minutes. One such study noted that a certain chemotherapy drug (Gemcitabine) is effective in eliminating double minutes from ovarian cancer cells [2]. The authors note that eliminating double minutes is important because it decreases the malignancy of cancer. However, there are few computational tools available for detecting double minute chromosomes. Raphael et al. [5] provide a method to reconstruct the tumor amplisome. They constructed a bacterial artificial chromosome (BAC) library from the MCF7 breast cancer cell line, and they reconstructed the amplisome of this cell line using end sequence profiling (ESP), which uses fragments of 100-300 kb in length. Using matched tumor and normal samples in NGS data, Sanborn et al. [6] provide methods that can reconstruct circular genome assemblies, including DMs and homogenously staining regions (HSRs). They used their method to reconstruct double minutes in tumors from patients with glioblastoma multiforme (GBM).

As mentioned by Raphael et al., concurrent analysis of the amplisome and genomic rearrangements is an important problem. In the context of double minute (DM) detection, we want to identify 1) contiguous amplified segments, and 2) the breakpoints that unite these contiguous segments. Identifying these breakpoints is analogous to the problem of detecting genomic rearrangements that are caused by large structural variants (SV), including deletions, translocations, inversions, and tandem duplications. Integrating SV breakpoint prediction with amplicon detection is essential to algorithmically discovering DMs.

**Figure 1:**
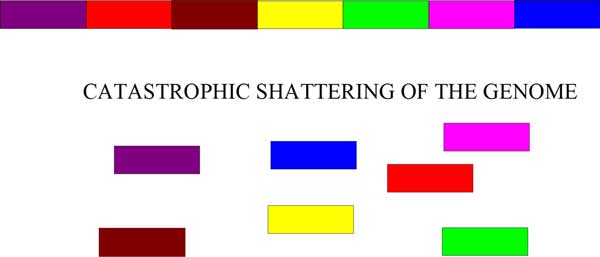
**Chromothripsis: the haphazard shattering of the genome during cancer development**.

**Figure 2:**
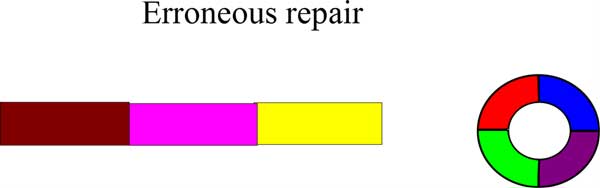
**The erroneous repair of the genome in Figure 1 leads to a circular double minute chromosome**.

Double minute chromosomes form during tumor development. There are several mechanisms that exist for the formation of double minutes. One of the mechanisms for their formation is a process known as *chromothripsis *[7], which means "genome shattering". Changes to the genome during cancer development can be gradual, but this phenomenon suggests that changes may also occur as a result of a single catastrophic event. Figure 1 illustrates the process of chromothripsis. A portion of the genome has haphazardly shattered as a consequence of cancer development. In Figure 2, erroneous DNA repair has taken place, which has led to the formation of a double minute chromosome. Chromothripsis is *not *a necessary condition for the formation of double minutes, but it is one mechanism under which it may occur. Another mechanism, known as the "episome" model, suggests that double minutes form when chromosomal segments are excised, circularized, and amplified [8]. Other mechanisms for DM formation have been proposed (such as those formed due to the breakage-fusion-bridge cycle [9]), though the exact nature of DM formation has yet to be determined in the general case.

Double minute chromosomes have two key properties. First, they are comprised of several distinct genomic segments. When these segments adjoin, their breakpoints resemble those of deletion, inversion, interchromosomal, and intrachromosomal rearrangements. These breakpoints can be discovered using a structural variation detection algorithm like BreakDancer [10]. The second distinctive property of double minutes is their propensity to amplify. Every segment comprising the double minute is an amplicon, and the copy numbers of these amplicons should be similar to one another.

**Figure 3:**
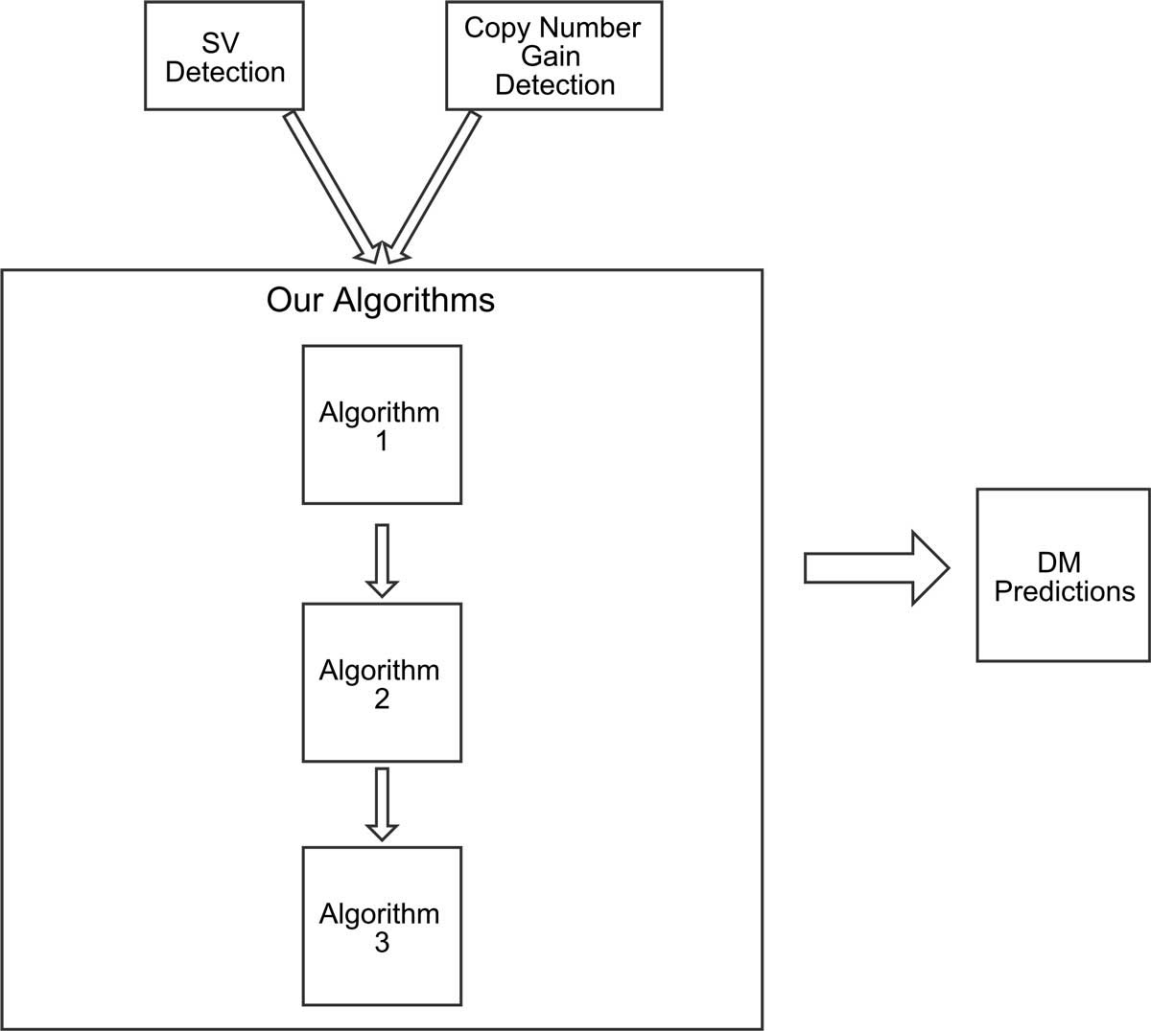
**Overview of the framework**. The algorithms require as input predictions from structural variant prediction programs and copy number prediction programs.

We present a framework for double minute detection that integrates copy number predictions and SV breakpoint predictions to locate DMs using NGS data. Our approach incorporates predictions from NGS-based copy number algorithms and SV algorithms. Using copy number and SV predictions, our framework incorporates them into a graph-based algorithm that can predict DMs in a tumor genome. Figure 3 diagrams the process of our method. We used RDXplorer [11] for our copy number variant analysis. For SV breakpoint prediction, we used BreakDancer [10] and Delly [12], which can identify SV breakpoints with high accuracy and specificity. We applied our framework to three simulated datasets containining DMs comprised of several amplicons. Our approach reconstructed several DMs with very high accuracy, while accurately discovering amplicons connected by rearrangement breakpoints. Our framework can be extended to any method that 1) predicts copy number gain and 2) discovers structural variant breakpoints.

## Methods

### Double minute discovery algorithms

**Figure 4:**
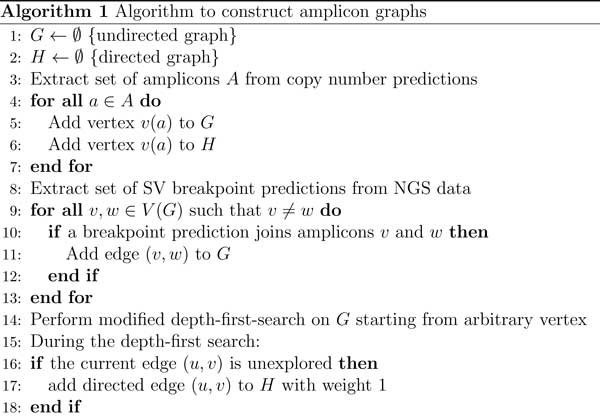
**Algorithm to build the amplicon graph *G *and auxiliary graph *H***.

**Figure 5:**
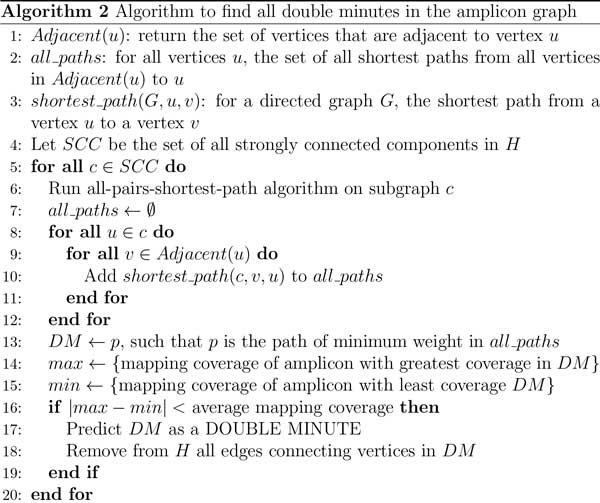
**Algorithm to find all double minutes that are represented in the amplicon graph**.

**Figure 6:**
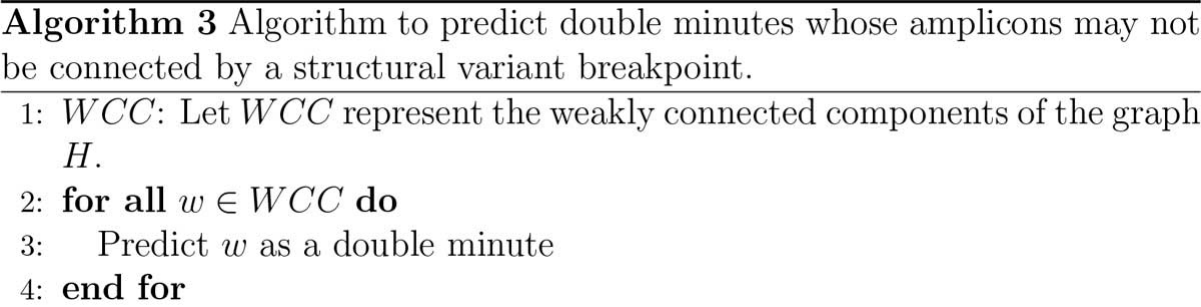
**Algorithm to find double minutes that may have been missed by algorithms 1 and 2**.

**Figure 7:**
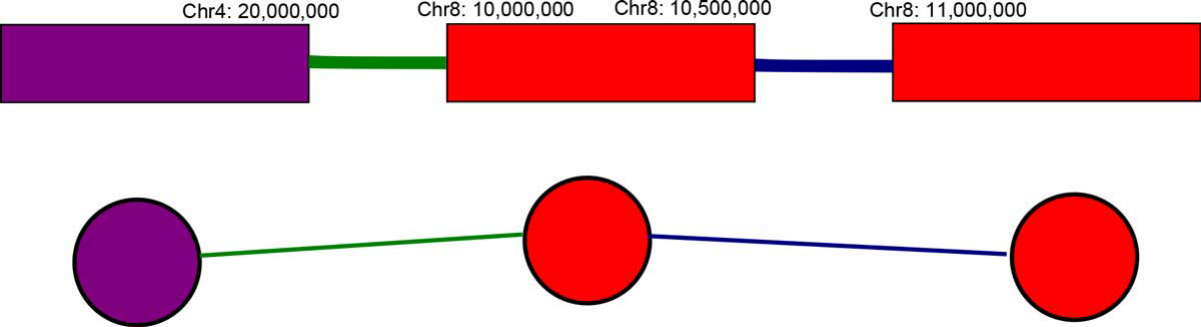
**Conversion of NGS breakpoints and amplicons to an amplicon graph**. The segments in the top image are amplicons with elevated copy number. The colored lines represent SV predictions for a translocation (green line), and a deletion (blue line). The corresponding amplicon graph is depicted in the bottom image. Vertices are amplicons and edges are NGS-based SV breakpoint predictions.

We take advantage of the distinctive properties of DMs to develop a unified approach to detect double minutes [13]. As stated previously, a method to detect complex SVs must have the ability to find basic SV breakpoints, and then to subsequently "group" breakpoints together as a single complex variant prediction. Our approach incorporates copy number variant predictions to find amplicons, and it takes a set of SV predictions from NGS data to find amplicons that are "linked" together, as would be seen in a double minute chromosome. These graph-based algorithms are provided in Figures 4, 5, and 6. The first algorithm constructs an *amplicon graph G*, and an auxiliary amplicon graph *H*. The graph *G *is undirected, and each vertex represents an amplicon that was discovered as per line 3. An edge connects two vertices (i.e., amplicons) in this graph if for an NGS-based SV prediction, its predicted breakpoints are proximal to the predicted breakpoints for each amplicon; we define "proximal" as being within *L *= *mean *+ *k *∗ *stdev*, where *mean *and *stdev *are the mean read pair mapping distance and its standard deviation, and *k *is a user-defined constant. Figure 7 shows how an amplicon graph is created from amplicon predictions and SV predictions.

#### Algorithm 1

Double minute chromosomes consist of consecutively-joined segments that are highly amplified. Our algorithms exploit the fact that double minutes are circular, and thus in the amplicon graph, the corresponding subgraph representing a true double minute should contain a simple path from some vertex back to itself. Lines 1-13 in Algorithm 1 are straightfoward. It simply builds the amplicon graph *G*, and it adds the same vertices in *G *to the auxiliary graph H. In line 13, we perform a modified depth-first search (DFS) on the graph *G *by keeping track of whether a currently explored edge has already been traversed. If not, then we add the corresponding directed edge to *H *with an edge weight of 1. This DFS is performed because we ultimately want our algorithm to identify cyclical subgraphs. If there is some simple path from a vertex *v *back to itself (excluding the trivial case where the path only contains *v*), a DFS will encounter a series of edges that represent a path from that vertex back to itself. While searching *G*, we add a directed edge to *H *if the current edge in the search is unexplored. This ensures that for the graph *G*, every vertex reachable from itself will have a corresponding directed path in *H *with a path to itself. If such a path can be identified, then it could indicate a possible double minute.

#### Algorithms 2 and 3

In Algorithm 2, the method processes the auxiliary graph *H *and determines whether or not it contains double minute chromosomes. It does this by first collecting all of the strongly connected components (SCC) in *H*, because a double minute is an SCC, or should at least be captured in a SCC. In line 5 of Algorithm 2, the method iterates through each SCC to see if it contains a double minute. It first runs an all-pairs-shortest-path algorithm on each retrieved SCC, and for each vertex *u *in an SCC, it searches for a shortest path from *v *to *u*, where *v *is a vertex adjacent to *u*. This step is performed because a double minute should contain a simple cycle from any vertex back to itself. We compute the shortest path for computational efficiency, as the longest simple path problem is NP-hard. However, we do not query the shortest path matrix for a path from *u *back to itself, because the shortest path from a node to itself is simply that node. To find a double minute, the method finds the shortest path cycle that minimizes the total edge weight. Thus, we assume that a double minute will be a cycle containing the fewest vertices (i.e., amplicons). This cycle is stored to *DM *in line 13. As previously stated, double minutes must consist of amplicons that have similar copy number. In NGS experiments, the copy number of a region in a donor genome is proportional to the sequence coverage in that region. Thus, each amplicon should also have similar mapping coverage. We check this condition by assessing the difference between the amplicon with the highest mapping coverage, and the amplicon with the lowest mapping coverage. This is performed in lines 15-17. If this difference is less than the average mapping coverage, then we predict the entire cycle in *DM *as a double minute.

**Figure 8:**
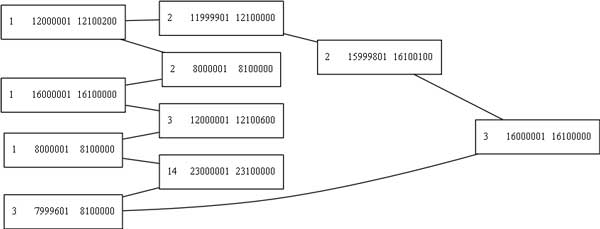
**A double minute chromosome found by our algorithms**. This is the double minute as it is represented in the amplicon graph *G*. Each node is an amplicon, and the edges are structural variant breakpoints that connect them. The nodes are annotated with the chromosome number and the start and end position of the amplicon. The graph image was rendered with GraphViz [17].

**Figure 9:**
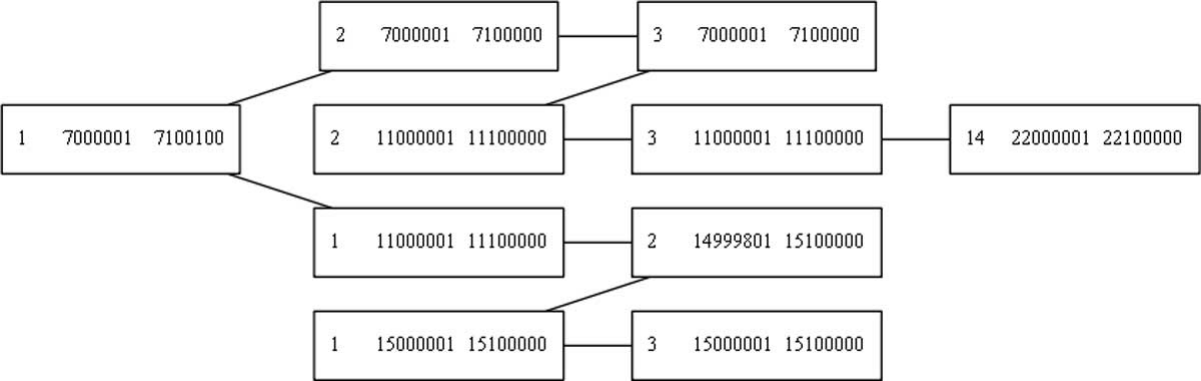
**A predicted DM whose circular structure was not captured in the data**. All 10 amplicons are present in this predicted double minute.

As shown in Figure 8, Algorithms 1 and 2 can reconstruct the circular nature of double minute chromosomes. Each of the amplicons (vertices) in the figure have similar copy number, which is expected for amplicons that belong to the same DM. However, some DMs may go undetected, because the SV and copy number signals may be insufficient to create a cycle in the auxiliary graph. Algorithm 3 accounts for double minutes whose circular nature is not captured by the datasets or by the SV and copy number programs. It does this by simply extracting the weakly connected components of the graph *H*. Figure 9 illustrates an acyclic predicted DM.

## Results

We tested our double minute prediction algorithm on three simulated NGS datasets. We created 20 double minutes, each comprised of 10 amplicons whose coordinates we chose beforehand. These regions were selected from chromosomes 1, 2, 3, and 14. The double minutes and their chosen coordinates are specified in Additional file 1. The selection of these chromosomes was arbitrary; any subset of chromosomes could have been selected for this experiment. From the coordinates, we extracted the corresponding FASTA sequences from the human reference genome build 37 (hg19). We then copied the sequences five times (5 copies), then two times (2 copies), then one time (1 copy), assigning each group of copied sequences to its own dataset. We then appended chromosomes 1, 2, 3, and 14 to each of the three datasets (5 copies, 2 copies, 1 copy). We then created simulated sequence reads (in FASTQ format) from each of these datasets using WGSim [14]. The reads were "sequenced" at 35X coverage with 100 bp reads and 400 bp fragment lengths, with a standard deviation of 50. The mutation rate was set to 0.001 and the indel rate was set to 0.15, which are default values. The default base error rate of 0.02 was used.

### Design

We aligned the aforementioned sequence reads to chromosomes 1, 2, 3 and 14 of the human reference genome using Bowtie2 [15]. To detect amplicons, we provided the resulting SAM file to RDXplorer, which is a program to detect copy number variants in NGS data [16]. This step is necessary to fulfill line 3 in Algorithm 1 (Figure 4). After acquiring the amplicons and applying a simple procedure to merge copy number predictions, we applied the BreakDancer and Delly algorithms to the alignments to uncover deletion, intrachromosomal translocation, interchromsomal translocation, and inversion breakpoints [10,12]. These types of rearrangements comprise the breakpoints that are seen in double minutes. After this step, we looked for all predicted double minutes that consisted of 7 or more amplicons, since we only wanted to predict a DM if many of its amplicons were linked together by our algorithms (we have 10 amplicons per DM). Real DMs can have dozens of amplicons, so this number is reasonable in the general case. A complete description of programs and settings used in our analysis is provided in Additional file 2.

### Experimental results

Figure 8 provides an example of a double minute that was reconstructed from the 5 copies dataset. Table 1 provides an overview of the sensitivity of our method to detect the synthetic double minutes when BreakDancer was used to detect structural variant breakpoints. Table 2 provides the results of the experiment when Delly was used to detect breakpoints.

**Table 1:** RDXplorer + BreakDancer.

Dataset	DMs detected
1 Copy	0/20

2 Copies	16/20

5 Copies	16/20

Summary of the double minutes found by our algorithms on each of the three datasets. There were 20 simulated double minutes in each dataset.

**Table 2:** RDXplorer + Delly.

Dataset	DMs detected
1 Copy	0/20

2 Copies	17/20

5 Copies	18/20

Summary of the double minutes found by our algorithms on each of the three datasets.

It should be noted that although BreakDancer, Delly, and RDXplorer were used for this experiment, our framework is applicable to any methods that call deletion, translocation, inversion, and duplication breakpoints, and any methods that predict copy number gains. Results from several programs may also be combined and provided as input to the framework. The method failed to return predictions for the 1 Copy dataset. This is not unexpected, as this dataset has the lowest read depth per DM, which decreases the ability of the framework to detect them. In reality, double minutes are highly amplified beyond just a single copy, so the framework is likely to have an ample signal to make predictions.

## Conclusion

We have presented a framework for detecting double minute chromosomes in next generation sequencing data. Our method accurately reconstructed double minutes whose signal was sufficiently represented in the structural variant and copy number predictions. Our method is also flexible; it can be applied to any SV and copy number resolution algorithms, thus giving a researcher the choice of methods to use in analysis. Future work will entail the use of real patient cancer datasets. Double minutes are present in many cancers, so using data from tumors would give better insights into the strengths and weaknesses of the method on real data.

Regarding limitations, our method currently does not attempt to measure the abundance of predicted DMs. Such a method would take into consideration the read depth of the amplicons that are predicted to comprise the double minute. Such information would be very helpful to a researcher who is attempting to assess the malignancy of a tumor that contains DMs. Future work will address such methods. Furthermore, the method currently looks for double minutes that are along a shortest path in the auxiliary graph. This was done for computational efficiency, but biologically, a double minute may exist along *any *path. We will explore this issue in subsequent publications. Lastly, we will consider more sophisticated methods of finding double minutes that may have been missed due to a lack of a clear signal in the data. With Algorithm 3, it is possible to have a predicted double minute that exhibits a bizarre graph structure (e.g. like a star graph), so we will explore more intelligent methods to address this problem that will take into account the circular nature of DMs.

## Competing interests

The authors declare that they have no competing interests.

## Authors' contributions

JL initiated the study. MH conceived of the idea and wrote the manuscript.

## Supplementary Material

Additional file 1 Describes the datasets and how they were generated. Click here for file

Additional file 2 Settings and parameters used for the structural variant and copy number prediction programs. Click here for file

## Declarations

The publication costs for the article were funded by the authors.

This article has been published as part of *BMC Genetics *Volume 16 Supplement 2, 2015: Selected articles from The 11th Annual Biotechnology and Bioinformatics Symposium (BIOT-2014): Genetics. The full contents of the supplement are available online at http://www.biomedcentral.com/bmcgenet/supplements/16/S2.
