# Supplementary material for: An integrative framework for the identification of double minute chromosomes using next generation sequencing data
Source: BMC Genet. 2015 Apr 23;16(Suppl 2):S1. doi: 10.1186/1471-2156-16-S2-S1 (PMC4423570; doi:10.1186/1471-2156-16-S2-S1)
Supplement: Additional file 1 — Describes the datasets and how they were generated. [file 1471-2156-16-S2-S1-S1.pdf]

An Integrative Framework for the Identification of Double Minute Chromosomes Using Next Generation Sequencing Data

Simulated Data Creation

WGSim Non-Default Settings

- -h: N
- -d: 400
- -s: 50
- -1: 100
- -2: 100
- -N: 139385615
    - This number was used to give us an average coverage of 35 reads per base.

Selected Breakpoint and Amplicon Coordinates

The coordinates chosen to comprise each DM are listed in the Appendix. Each grouping of coordinates is one double minute, and each row is an amplicon (with the exception of the two chr14 rows, which together form a single amplicon). The rearrangement breakpoints are formed by the fusing of consecutive amplicons. For each row, we put a "+" if the segment lies on the 5'-3' strand (with respect to the p-arm telomere), and a "-" if the segment is on the 3'-5' strand. For each double minute, the two chr14 entries denote the same amplicon; this was done to facilitate sequence extraction using the FastaFromBed tool in the BEDTools suite [1].

After each sequence corresponding to each row was extracted, it was copied {1,2,5} times and appended to the {1,2,5}.fa FASTA files. Each of these three FASTA files also contained a full copy of chromosomes 1,2,3 and 14. After the creation of these FASTA files, we simulated the creation of FASTQ sequence reads using WGSim, per the parameters described above. Once the FASTQ files were created, they were aligned to hg19 chromosomes 1,2,3, and 14 as described in the "Programs and Settings.docx" supplement.

APPENDIX

Coodinates Chosen for Simulated Double Minutes

**DM 1**			
chr14	20050000	20100000	+
chr2	9000000	9100000	+
chr3	9000000	9100000	-
chr1	13000000	13100000	-
chr2	13000000	13100000	-
chr1	9000000	9100000	+
chr3	13000000	13100000	-
chr1	5000000	5100000	-
chr2	5000000	5100000	-
chr3	5000000	5100000	-
chr14	20000000	20049999	+
**DM 2**			
chr14	20250000	20300000	+
chr2	9200000	9300000	+
chr3	9200000	9300000	-
chr1	9200000	9300000	-
chr1	5200000	5300000	+
chr2	13200000	13300000	+
chr2	5200000	5300000	-
chr3	13200000	13300000	-
chr3	5200000	5300000	-
chr1	13200000	13300000	-
chr14	20200000	20249999	+
**DM 3**			
chr14	20450000	20500000	+
chr3	9400000	9500000	+
chr3	5400000	5500000	-
chr1	9400000	9500000	+
chr2	9400000	9500000	+
chr3	13400000	13500000	+
chr1	13400000	13500000	+
chr1	5400000	5500000	-
chr2	5400000	5500000	+
chr2	13400000	13500000	-
chr14	20400000	20449999	+
**DM 4**			
chr14	20650000	20700000	+

chr1	9600000	9700000	+
chr2	5600000	5700000	-
chr1	5600000	5700000	+
chr3	13600000	13700000	-
chr1	13600000	13700000	+
chr3	5600000	5700000	+
chr2	9600000	9700000	-
chr3	9600000	9700000	+
chr2	13600000	13700000	-
chr14	20600000	20649999	+
**DM 5**			
chr14	20850000	20900000	+
chr1	5800000	5900000	-
chr1	9800000	9900000	-
chr2	13800000	13900000	+
chr3	9800000	9900000	-
chr3	13800000	13900000	+
chr2	5800000	5900000	-
chr2	9800000	9900000	+
chr3	5800000	5900000	+
chr1	13800000	13900000	-
chr14	20800000	20849999	+
**DM 6**			
chr14	21050000	21100000	+
chr3	6000000	6100000	-
chr3	10000000	10100000	-
chr1	10000000	10100000	+
chr2	10000000	10100000	-
chr2	6000000	6100000	-
chr2	14000000	14100000	+
chr1	6000000	6100000	+
chr1	14000000	14100000	-
chr3	14000000	14100000	+
chr14	21000000	21049999	+
**DM 7**			
chr14	21250000	21300000	+
chr3	10200000	10300000	+
chr1	6200000	6300000	-
chr3	6200000	6300000	+
chr2	14200000	14300000	+
chr3	14200000	14300000	+

chr2	6200000	6300000	-
chr2	10200000	10300000	+
chr1	10200000	10300000	+
chr1	14200000	14300000	-
chr14	21200000	21249999	+
**DM 8**			
chr14	21450000	21500000	+
chr1	14400000	14500000	-
chr1	6400000	6500000	+
chr2	6400000	6500000	+
chr3	14400000	14500000	+
chr2	14400000	14500000	+
chr3	6400000	6500000	-
chr3	10400000	10500000	-
chr1	10400000	10500000	+
chr2	10400000	10500000	+
chr14	21400000	21449999	+
**DM 9**			
chr14	21650000	21700000	+
chr2	14600000	14700000	-
chr1	10600000	10700000	+
chr3	6600000	6700000	-
chr2	6600000	6700000	+
chr3	14600000	14700000	-
chr1	6600000	6700000	-
chr3	10600000	10700000	-
chr1	14600000	14700000	-
chr2	10600000	10700000	-
chr14	21600000	21649999	+
**DM 10**			
chr14	21850000	21900000	+
chr2	10800000	10900000	-
chr3	10800000	10900000	-
chr2	14800000	14900000	+
chr3	14800000	14900000	+
chr1	14800000	14900000	+
chr1	6800000	6900000	+
chr3	6800000	6900000	-
chr2	6800000	6900000	+
chr1	10800000	10900000	+
chr14	21800000	21849999	+

**DM 11**			
chr14	22050000	22100000	+
chr3	11000000	11100000	+
chr2	11000000	11100000	-
chr3	7000000	7100000	+
chr2	7000000	7100000	+
chr1	7000000	7100000	+
chr1	11000000	11100000	+
chr2	15000000	15100000	+
chr1	15000000	15100000	+
chr3	15000000	15100000	-
chr14	22000000	22049999	+
**DM 12**			
chr14	22250000	22300000	+
chr2	7200000	7300000	-
chr2	11200000	11300000	-
chr1	11200000	11300000	-
chr1	15200000	15300000	-
chr1	7200000	7300000	+
chr2	15200000	15300000	+
chr3	11200000	11300000	+
chr3	7200000	7300000	+
chr3	15200000	15300000	-
chr14	22200000	22249999	+
**DM 13**			
chr14	22450000	22500000	+
chr1	7400000	7500000	+
chr2	15400000	15500000	+
chr1	15400000	15500000	+
chr2	11400000	11500000	+
chr3	11400000	11500000	+
chr3	7400000	7500000	+
chr1	11400000	11500000	+
chr3	15400000	15500000	-
chr2	7400000	7500000	+
chr14	22400000	22449999	+
**DM 14**			
chr14	22650000	22700000	+
chr3	11600000	11700000	-
chr2	7600000	7700000	-
chr1	11600000	11700000	-

chr2	11600000	11700000	+
chr1	15600000	15700000	-
chr2	15600000	15700000	-
chr3	7600000	7700000	+
chr3	15600000	15700000	+
chr1	7600000	7700000	-
chr14	22600000	22649999	+
**DM 15**			
chr14	22850000	22900000	+
chr1	15800000	15900000	+
chr2	11800000	11900000	-
chr3	7800000	7900000	+
chr3	11800000	11900000	+
chr2	15800000	15900000	-
chr1	11800000	11900000	-
chr3	15800000	15900000	+
chr2	7800000	7900000	-
chr1	7800000	7900000	-
chr14	22800000	22849999	+
**DM 16**			
chr14	23050000	23100000	+
chr1	8000000	8100000	-
chr3	12000000	12100000	-
chr1	16000000	16100000	-
chr2	8000000	8100000	-
chr1	12000000	12100000	-
chr2	12000000	12100000	+
chr2	16000000	16100000	+
chr3	16000000	16100000	-
chr3	8000000	8100000	+
chr14	23000000	23049999	+
**DM 17**			
chr14	23250000	23300000	+
chr3	12200000	12300000	-
chr1	12200000	12300000	-
chr3	8200000	8300000	-
chr1	8200000	8300000	-
chr2	16200000	16300000	+
chr2	12200000	12300000	-
chr3	16200000	16300000	+
chr1	16200000	16300000	-

chr2	8200000	8300000	+
chr14	23200000	23249999	+
**DM18**			
chr14	23450000	23500000	+
chr2	8400000	8500000	+
chr2	16400000	16500000	-
chr3	16400000	16500000	-
chr3	12400000	12500000	-
chr2	12400000	12500000	+
chr1	16400000	16500000	-
chr1	12400000	12500000	-
chr1	8400000	8500000	+
chr3	8400000	8500000	+
chr14	23400000	23449999	+
**DM 19**			
chr14	23650000	23700000	+
chr3	12600000	12700000	-
chr1	8600000	8700000	+
chr2	12600000	12700000	+
chr2	16600000	16700000	-
chr3	8600000	8700000	-
chr1	16600000	16700000	+
chr3	16600000	16700000	-
chr1	12600000	12700000	-
chr2	8600000	8700000	-
chr14	23600000	23649999	+
**DM 20**			
chr14	23850000	23900000	+
chr3	12800000	12900000	-
chr2	16800000	16900000	+
chr2	8800000	8900000	-
chr2	12800000	12900000	+
chr1	16800000	16900000	-
chr1	12800000	12900000	+
chr3	8800000	8900000	-
chr3	16800000	16900000	+
chr1	8800000	8900000	-
chr14	23800000	23849999	+

REFERENCES

[1] Quinlan AR and Hall IM, 2010. BEDTools: a flexible suite of utilities for comparing genomic features. Bioinformatics. 26, 6, pp. 841–842.
