# Supplementary material for: An integrative framework for the identification of double minute chromosomes using next generation sequencing data
Source: BMC Genet. 2015 Apr 23;16(Suppl 2):S1. doi: 10.1186/1471-2156-16-S2-S1 (PMC4423570; doi:10.1186/1471-2156-16-S2-S1)
Supplement: Additional file 2 — Settings and parameters used for the structural variant and copy number prediction programs. [file 1471-2156-16-S2-S1-S2.pdf]

An Integrative Framework for the Identification of Double Minute Chromosomes Using Next Generation Sequencing Data

Programs and Settings

Alignment with Bowtie2 version 2.2.3

1. The Bowtie2 index files for human reference genome (hg19) chromosomes 1,2,3, and 14 were generated with bowtie2-build. Default parameters were used.
2. For alignment with Bowtie2, default parameters were used except for the following parameters:
    - The “--local" option was set.
    - –X: 600

Predicting Amplicons with RDXplorer 2.0

Other than to specify chromosomes 1,2,3, and 14, all default parameters were used.

Merging of Amplicons

Before sending the copy number predictions to our algorithms, we merged amplicons (predicted copy number gains) that were separated by 10,000 bases or less.

Predicting Structural Variant Breakpoints with BreakDancer 1.1

1. Running the configuration script (bam2cfg.pl)
    - Non-default parameters used
        - q: 30
2. Running the main program (breakdancer_max)
    - c: 4
    - q: 30
    - r: 4

Predicting Structural Variant Breakpoints with Delly 0.6.1

Delly was configured to discover the following kinds of structural variants:

- Deletions (DEL flag)

- Translocations (TRA flag)
- Duplications (DUP flag)
- Inversions (INV flag)

The following non-default parameters were used:

- -x: human.hg19.excl.tsv
    - This file was provided by the Delly authors. It allows the program to exclude the telomeric and centromeric regions from analysis.
- -q: 30

Discovering Double Minutes with DMFinder 1.0

- All default parameters used
